# Immunogenicity of Inactivated Varicella Zoster Vaccine in Autologous Hematopoietic Stem Cell Transplant Recipients and Patients With Solid or Hematologic Cancer

**DOI:** 10.1093/ofid/ofaa172

**Published:** 2020-06-02

**Authors:** Michael J Boeckh, Ann M Arvin, Kathleen M Mullane, Luis H Camacho, Drew J Winston, Vicki A Morrison, Kimberly Hurtado, Jessie Durrand Hall, Lei Pang, Shu-Chih Su, Susan S Kaplan, Paula W Annunziato, Zoran Popmihajlov, V212 Protocol 001 Trial Group and V212 Protocol 011 Trial Group

**Affiliations:** 1 Vaccine and Infectious Disease & Clinical Research Divisions, Fred Hutchinson Cancer Research Center, Seattle, Washington, USA; 2 Microbiology & Immunology Departments, Stanford University School of Medicine, Stanford, California, USA; 3 Department of Medicine, University of Chicago, Chicago, Illinois, USA; 4 Medical Oncology, Oncology Consultants, Houston, Texas, USA; 5 Department of Medicine, University of California Los Angeles Medical Center, Los Angeles, California, USA; 6 Hematology Oncology Division, Hennepin County Medical Center, University of Minnesota, Minneapolis, Minnesota, USA; 7 Merck & Co., Inc., Kenilworth, New Jersey, USA

**Keywords:** cell-mediated immunity, humoral immunity, immunocompromised adults, immunogenicity, inactivated zoster vaccine

## Abstract

**Background:**

In phase 3 trials, inactivated varicella zoster virus (VZV) vaccine (ZV_IN_) was well tolerated and efficacious against herpes zoster (HZ) in autologous hematopoietic stem cell transplant (auto-HSCT) recipients and patients with solid tumor malignancies receiving chemotherapy (STMc) but did not reduce HZ incidence in patients with hematologic malignancies (HMs). Here, we describe ZV_IN_ immunogenicity from these studies.

**Methods:**

Patients were randomized to ZV_IN_ or placebo (4 doses). Immunogenicity was assessed by glycoprotein enzyme-linked immunosorbent assay (gpELISA) and VZV interferon (IFN)-γ enzyme-linked immunospot (ELISPOT) assay in patients receiving all 4 doses without developing HZ at the time of blood sampling.

**Results:**

Estimated geometric mean fold rise ratios (ZV_IN_/placebo) by gpELISA and IFN-y ELISPOT ~28 days post–dose 4 were 2.02 (95% confidence interval [CI], 1.53–2.67) and 5.41 (95% CI, 3.60–8.12) in auto-HSCT recipients; 1.88 (95% CI, 1.79–1.98) and 2.10 (95% CI, 1.69–2.62) in patients with STMc; and not assessed and 2.35 (95% CI, 1.81–3.05) in patients with HM.

**Conclusions:**

ZV_IN_ immunogenicity was directionally consistent with clinical efficacy in auto-HSCT recipients and patients with STMc even though HZ protection and VZV immunity were not statistically correlated. Despite a lack of clinical efficacy in patients with HM, ZV_IN_ immunogenicity was observed in this population. Immunological results did not predict vaccine efficacy in these 3 populations.

**Clinical trial registration:**

NCT01229267, NCT01254630.

Cell-mediated immunity plays a critical role in the containment of varicella zoster virus (VZV), preventing the reactivation of VZV and subsequent onset of herpes zoster (HZ) [1]. Immunocompromised individuals, such as patients who have undergone autologous hematopoietic stem cell transplant (auto-HSCT) or patients with malignancies, are at ~3–18-fold increased risk of HZ infection compared with immunocompetent patients, depending on the nature of the underlying condition [2–6]. The reported incidence of HZ in auto-HSCT recipients, despite antiviral prophylaxis, ranges from 62 of 1000 person-years (PYs), based on a large retrospective analysis [4], to 92 of 1000 PYs, based on a recent phase 3 randomized clinical trial [7]. For patients with solid tumor malignancies receiving chemotherapy (STMc), reports of HZ incidence range from 15 of 1000 PYs [3] to 19 of 1000 PYs [8]. For patients with hematologic malignancies (HMs), HZ incidence is reported to be 31 of 1000 PYs [2, 6, 8]. In comparison, HZ incidence in the the general adult population is 5 of 1000 PYs [9].

In immunocompromised patients such as these, HZ causes significant morbidity, including pain, post-herpetic neuralgia, HZ ophthalmicus, visceral organ involvement, hospitalization, and occasional mortality [10–13]. Additionally, secondary bacterial infection, such as streptococcal or staphylococcal superinfections, can complicate HZ rash and, although complete healing of HZ rash typically occurs within 2 to 4 weeks, pigmentation changes and scarring may be permanent [13, 14].

The live attenuated VZV vaccine is contraindicated in immunocompromised patients [15]; therefore, an inactivated VZV vaccine (ZV_IN_) was investigated as a preventive option for immunocompromised patients. Proof-of-concept studies and a phase 1 trial using a heat-treated ZV_IN_ demonstrated immunogenicity and safety in auto-HSCT recipients and patients with STMc or HM through 28 days post–dose 4 following a 4-dose regimen administered ~30 days apart [16–18]. Subsequently, phase 1 and 2 trials using ZV_IN_ inactivated by gamma irradiation confirmed immunogenicity and safety in patients with HM receiving anti-CD20 monoclonal antibodies and in adults with autoimmune disease receiving immunosuppressive therapy, respectively [19, 20].

Primary safety and efficacy results from 2 phase 3 trials (V212-001 and V212-011) demonstrated that ZV_IN_ was well tolerated, with the incidence of HZ and HZ-related complications significantly reduced in auto-HSCT recipients and patients with STMc but not in patients with HM [7, 8]. In auto-HSCT recipients, the estimated vaccine efficacy of ZV_IN_ against HZ (VE_HZ_) was 63.8% (95% confidence interval [CI], 48.4%–74.6%) [7]; in patients with STMc, the estimated VE_HZ_ was 63.6% (97.5% CI, 36.4%–79.1%) [8]. Immunogenicity was assessed as an exploratory end point in these 2 phase 3 trials, with the results presented here.

## METHODS

### Trial Designs

V212-001 (NCT01229267) and V212-011 (NCT01254630) were phase 3, randomized, double-blind, placebo-controlled multicenter trials that evaluated the safety, tolerability, efficacy, and immunogenicity of ZV_IN_ for the prevention of HZ and HZ-related complications in auto-HSCT recipients (V212-001) and in patients with STMc or HM (V212-011). V212-001 was conducted between December 2010 and December 2015; V212-011 was conducted between June 2011 and April 2017. Ethical approval was obtained from the institutional review board at each trial site, and written informed consent was obtained from each participant before trial entry. The V212-001 and V212-011 protocols have been previously described [7, 8]. The studies were conducted in accordance with the principles of Good Clinical Practice. Patients were monitored for clinical signs and symptoms of HZ and HZ-related complications through the entire trial period. HZ was diagnosed primarily by polymerase chain reaction [7, 8].

### Trial Population

Trials included males and females aged 18 years or older with a history of varicella infection or seropositivity for VZV antibody. V212-001 included participants scheduled to receive auto-HSCT for treatment of lymphoma, other malignancies, or any nonmalignant conditions within 60 days of enrollment. The exclusion criteria in V212-001 included underlying malignancies other than Hodgkin lymphoma associated with >2 disease relapses, planned tandem transplantation, and intended antiviral prophylaxis for more than 6 months after transplantation. V212-011 included patients with STMc or HM who were not likely to undergo HSCT and who were receiving a cytotoxic or immunosuppressive chemotherapy regimen. Patients with HM who were ≥50 years of age and not in remission were eligible, regardless of whether they were receiving chemotherapy. Common exclusion criteria in both trials were history of HZ within 1 year of enrollment and prior or expected receipt of any VZV vaccine. The exclusion criteria in V212-011 included current/expected receipt of long-term (>4 weeks) antiviral prophylaxis against HSV, VZV, or CMV.

### Treatment Administration

In these trials, patients were randomly allocated to receive gamma-irradiated ZV_IN_ or placebo, administered in a 4-dose regimen ~30 days apart [7, 8]. For more details on the vaccine, see the Supplementary Methods. Auto-HSCT recipients received dose 1 ~30 days (60 to 5 days) before HSCT. Doses 2 through 4 were administered 30, 60, and 90 days after HSCT. Both patients with STMc and patients with HM received dose 1 of ZV_IN_ or placebo at the time of enrollment (day 1). Doses 2 through 4 were administered ~30 days after each previous dose. Among patients receiving cyclic chemotherapy, dose 1 of ZV_IN_ or placebo was administered ~5 days before any chemotherapy dose in the cycle. Doses 2 through 4 were administered ~20 to 40 days after the previous dose of vaccine or placebo; specifically, ZV_IN_ or placebo had to be administered ~5 days before the upcoming chemotherapy dose. To complete the studies, patients had to have completed the studies’ close-out questionnaires at the end of the safety follow-up period; these were administered over the phone. In V212-011, the HM group was discontinued due to statistical evidence of futility shown at a planned interim analysis.

### Immunogenicity Measurements

Immunogenicity analyses were exploratory (no prespecified statistical hypotheses were tested) and were conducted in the per-protocol immunogenicity population. In both trials, the per-protocol immunogenicity population included patients who received all 4 doses and did not have HZ before blood sampling. For patients who received treatments interfering with measurements of VZV-specific antibody response (including those receiving immunoglobulin therapy) or who received medications interfering with B-cell function, the measurements at corresponding time points and thereafter were excluded from the immunogenicity analysis conducted by glycoprotein enzyme-linked immunosorbent assay (gpELISA). Patients who received immunoglobulin therapies were included in the immunogenicity analysis conducted by VZV interferon (IFN)-γ enzyme-linked immunospot (IFN-γ ELISPOT) assay because they did not interfere with T-cell function.

VZV-specific antibody responses were measured by gpELISA assay [21] in auto-HSCT recipients and in patients with STMc. This antibody assay was not conducted in patients with HM because the nature of the disease and treatments could have biased the test results, based on the results from previous phase 1 gpELISA data in patients with HM [17]. Cell-mediated immune responses were measured by VZV IFN-γ ELISPOT assay [22] in subsets of auto-HSCT recipients, patients with STMc, and patients with HM. The VZV ELISPOT assay detected IFN-γ–secreting cells from peripheral blood mononuclear cells stimulated with VZV before and after vaccination. For immune responses measured by gpELISA, end points were the GMT and geometric mean fold rise (GMFR). For immune responses measured by VZV IFN-γ ELISPOT assay, end points were geometric mean count (GMC) and GMFR.

In auto-HSCT recipients, blood samples for immunogenicity analyses were collected on day 1 (before dose 1), ~28 days (21–35 days) post–dose 3, ~28 days (28–60 days) post–dose 4, and annually post–dose 4 until the end of the trial. In patients with STMc and HM, blood samples for immunogenicity analyses were collected on day 1 (before dose 1) and ~28 days (28–60 days) post–dose 4. PPD Vaccines and Biologics, LLC (Wayne, PA, USA), performed the gpELISA on serum samples, and ViraCor-IBT Laboratories, Inc. (Lenexa, KS, USA), performed the IFN-γ ELISPOT assays on peripheral blood mononuclear cell samples.

### Statistical Analyses

A linear mixed longitudinal model was used on the natural log-transformed antibody titers for the comparison of GMTs between ZV_IN_ and placebo recipients across the time points after vaccination. This longitudinal regression approach allowed for comparison of postvaccination antibody titers between the groups, adjusting for prevaccination antibody titer in the presence of incomplete data [23]. The model incorporated treatment group, visit, age (for V212-001: <50 vs ≥ 50 years; for V212-011: continuous variable), and treatment group-by-visit interaction. The fold-differences between the ZV_IN_ and placebo recipients and the corresponding 95% CIs at the visits were obtained from this model.

A Cox regression model was used for ZV_IN_ and placebo recipients, with immune responses measured by gpELISA at prespecified time points as covariates, to evaluate the association between immune responses and risk of HZ. The GMT and GMFR were also summarized at these time points by treatment group and HZ outcome (patients who developed confirmed HZ during the trial vs patients who did not).

A time-varying Cox proportional hazards model was used to estimate the relationship between HZ occurrence and gpELISA titers among ZV_IN_ and placebo recipients. The gpELISA titers were used as the time-dependent covariate to obtain a risk ratio for HZ per unit increase in the titer. Using the natural log-scale of GMC, analyses of the comparison of GMC between treatment groups and evaluation of the association between GMC and HZ risk were performed similarly to the analyses and evaluations for gpELISA.

## RESULTS

### Demographics

Overall, 1257 auto-HSCT recipients were randomized to ZV_IN_ (n = 560) or placebo (n = 564) (Supplementary Figure 1) [7], 2712 patients with STMc were randomized to ZV_IN_ (n = 1348) or placebo (n = 1364) (Supplementary Figure 2) [8], and 2573 patients with HM were randomized to ZV_IN_ (n = 1288) or placebo (n = 1285) (Supplementary Figure 2) [8]. More than 80% of patients (auto-HSCT recipients, 83%; patients with STMc, 87%; patients with HM, 90%) received all 4 doses. More than 70% of patients in each treatment group were older than 50 years (auto-HSCT recipients, 72%; patients with STMc, 77%–78%; patients with HM, 82%–83%). Among auto-HSCT recipients and patients with HM, the majority were male (auto-HSCT recipients, 64%; patients with HM, 59%). In contrast, most patients with STMc were female (64%–65%).

The most common primary diagnoses were myeloma among auto-HSCT recipients, breast cancer among patients with STMc, and chronic lymphocytic leukemia among patients with HM (Supplementary Table 1) [7, 8]. The most common concomitant medications included systemic antibacterial agents in auto-HSCT recipients, antineoplastic agents in patients with STMc, and analgesics in patients with HM (Supplementary Table 1) [8].

### VZV-Specific Antibody Response by Glycoprotein ELISA

**Figure 1.**
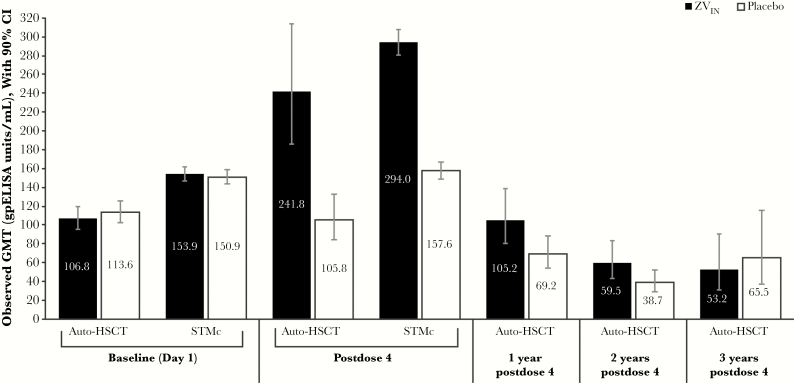
VZV-specific antibody observed response by gpELISA in auto-HSCT recipients and patients with STMc (per-protocol population). Abbreviations: auto-HSCT, autologous hematopoietic stem cell transplant; gpELISA, glycoprotein enzyme-linked immunosorbent assay; STMc, solid tumor malignancies receiving chemotherapy; ZV_IN_, inactivated varicella zoster.

The observed GMT values in the ZV_IN_ and placebo groups at baseline and the time points examined in auto-HSCT recipients and patients with STMc are shown in Figure 1 [7, 8]. In auto-HSCT recipients, the estimated GMFR ratio (ZV_IN_/placebo) was 2.02 (95% CI, 1.53–2.67) at ~28 days post–dose 4, 1.30 (95% CI, 0.99–1.71) at 1 year post–dose 4, and 1.34 (95% CI, 0.95–1.87) at 2 years post–dose 4 (Table 1). At 1 and 2 years post–dose 4, estimated VZV antibody responses remained slightly elevated compared with baseline levels (Table 1). Similarly, in patients with STMc, the estimated GMFR ratio was 1.88 (95% CI, 1.79–1.98) at ~28 days post–dose 4 (Table 1).

**Table 1.** VZV-Specific Antibody Estimated Response by gpELISA in Auto-HSCT Recipients and Patients With STMc (Per-Protocol Population)

gpELISA, Units/mL^a^			ZV_IN_		Placebo		GMFR Fold Difference^b^ for ZV_IN_/Placebo (95% CI)
			No.	Estimated Response^a^	No.	Estimated Response^a^	
Auto-HSCT	Number of patients vaccinated: ZV_IN_, 554; placebo, 556						
	~28 days post–dose 4	GMT GMFR^c^	377	218.0 1.96	387	107.9 0.97	2.02 (1.53–2.67)
	1 year post–dose 4	GMT GMFR^c^	377	102.4 0.92	387	78.7 0.71	1.30 (0.99–1.71)
	2 years post–dose 4	GMT GMFR^c^	377	67.0 0.60	387	50.2 0.45	1.34 (0.95–1.87)
STMc	Number of patients vaccinated: ZV_IN_, 1326; placebo, 1349						
	~28 days post–dose 4	GMT GMFR^c^	1266	295.5 1.94	1299	157.0 1.03	1.88 (1.79–1.98)

Abbreviations: auto-HSCT, autologous hematopoietic stem cell transplant; GMFR, geometric mean fold rise; gpELISA, glycoprotein enzyme-linked immunosorbent assay; No., number of patients contributing to the immunogenicity analysis; STMc, solid tumor malignancies receiving chemotherapy; ZV_IN,_ inactivated varicella zoster.

^a^Results for the gpELISA are reported as concentration of antibody in gpELISA units/mL.

^b^Calculated based on longitudinal regression model (adjusting for prevaccination immunogenicity level in the presence of incomplete data), with treatment group, visit, and treatment group-by-visit as covariates.

^c^From day 1.

### VZV-Specific Cell-Mediated Response by IFN-y Enzyme-Linked Immunospot Assay

**Figure 2.**
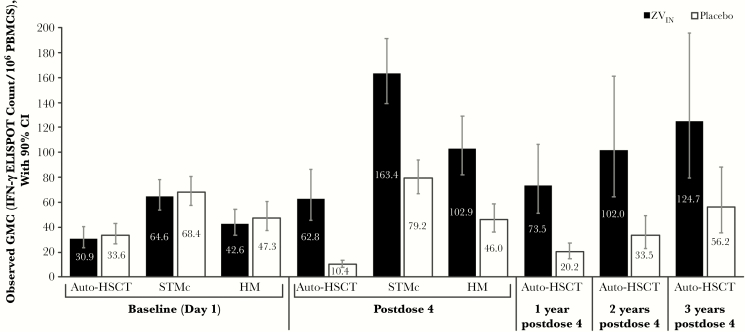
VZV-specific cell-mediated observed immune response by interferon (IFN)-γ ELISPOT assay in auto-HSCT recipients, patients with STMc, and patients with HM (per-protocol population). Abbreviations: auto-HSCT, autologous hematopoietic stem cell transplant; GMC, geometric mean count; HM, hematologic malignancies; IFN-γ ELISPOT, IFN-γ enzyme-linked immunospot; PBMCs, peripheral blood mononuclear cells; STMc, solid tumor malignancies receiving chemotherapy; ZV_IN_, inactivated varicella zoster.

The observed GMC values in the ZV_IN_ and placebo groups at baseline and at time points examined in auto-HSCT recipients and patients with STMc and HM are shown in Figure 2 [7, 8]. In auto-HSCT recipients, the estimated GMFR ratio (ZV_IN_/placebo) was 5.41 (95% CI, 3.60–8.12) at ~28 days post–dose 4, 4.12 (95% CI, 2.62–6.47) at 1 year post–dose 4, and 3.32 (95% CI, 1.90–5.82) at 2 years post–dose 4 (Table 2). ZV_IN_ recipients achieved an estimated GMFR from a baseline value of 1.85 at ~28 days post–dose 4, which increased up to 3.32 at 2 years post–dose 4. In placebo recipients, the estimated GMC values at ~28 days post–dose 4 and at 1 year post–dose 4 were lower than baseline values, and only at the 2-year post–dose 4 time point were estimated GMC values comparable with baseline. In patients with STMc and HM, the estimated GMFR ratios (ZV_IN_ /placebo) were 2.10 (95% CI, 1.69–2.62) and 2.35 (95% CI, 1.81–3.05), respectively, at ~28 days post–dose 4 (Table 2). Among ZV_IN_ recipients at ~28 days post–dose 4, estimated GMC values were highest in patients with STMc and lowest in auto-HSCT recipients.

**Table 2.** VZV-Specific Cell-Mediated Estimated Immune Response by IFN-γ ELISPOT Assay in Auto-HSCT Recipients, Patients With STMc, and Patients With HM (Per-Protocol Population)

IFN-γ ELISPOT Assay Count/10^6^ PBMCs^a^			ZV_IN_		Placebo		GMFR Fold Difference^b^ for ZV_IN_/Placebo (95% CI)
			No.	Estimated Response^b^	No.	Estimated Response^b^	
Auto-HSCT	Number of patients vaccinated: ZV_IN_, 186; placebo, 181						
	~28 days post–dose 4	GMC GMFR^c^	168	62.3 1.85	171	11.5 0.34	5.41 (3.60–8.12)
	1 year post–dose 4	GMC GMFR^c^	168	83.1 2.47	171	20.2 0.60	4.12 (2.62–6.47)
	2 years post–dose 4	GMC GMFR^c^	168	111.6 3.32	171	33.6 1.00	3.32 (1.90–5.82)
STMc	Number of patients vaccinated: ZV_IN_, 232; placebo, 246						
	~28 days post–dose 4	GMC GMFR^c^	208	163.2 2.46	221	77.6 1.17	2.10 (1.69–2.62)
HM	Number of patients vaccinated: ZV_IN_, 231; placebo, 247						
	~28 days post–dose 4	GMC GMFR^c^	187	97.5 2.22	183	41.5 0.94	2.35 (1.81–3.05)

Abbreviations: auto-HSCT, autologous hematopoietic stem cell transplant; IFN-γ ELISPOT, interferon-γ enzyme-linked immunospot; GMC, geometric mean count; GMFR, geometric mean fold rise; HM, hematologic malignancies; No., number of patients contributing to the immunogenicity analysis; PBMCs, peripheral blood mononuclear cells; STMc, solid tumor malignancies receiving chemotherapy; ZV_IN_, inactivated varicella zoster.

^a^Results from the IFN-γ ELISPOT assay are expressed as the frequency of spot-forming cells per million PBMCs.

^b^Calculated based on longitudinal regression model (adjusting for prevaccination immunogenicity level in the presence of incomplete data), with treatment group, visit, and treatment group-by-visit as covariates.

^c^From day 1.

### Association Between Immune Response and Risk of Herpes Zoster

Post–dose 4 gpELISA and VZV IFN-γ ELISPOT assay results were available for a small number of ZV_IN_ recipients who subsequently developed HZ (Tables 3 and 4). Among auto-HSCT recipients who received ZV_IN_, gpELISA GMT values at ~28 days post–dose 4 were lower in the group that developed HZ than in the group that did not, although CIs were broad and overlapping (Table 3). In patients with STMc, GMT values at ~28 days post–dose 4 were generally similar among those who did and did not develop HZ (Table 3). For both the auto-HSCT recipients and patients with HM who received ZV_IN_, VZV IFN-γ ELISPOT assay GMC values at ~28 days post–dose 4 were lower in the group that developed HZ than the group that did not, albeit with overlapping CIs (Table 4). Surprisingly, patients with STMc who developed HZ had high VZV IFN-γ ELISPOT assay GMC values at ~28 days post–dose 4, which were higher than GMC values among patients with STMc who did not develop HZ (Table 4). A statistical correlation was not found between HZ protection and VZV gpELISA response in auto-HSCT recipients and patients with STMc (Table 5). In addition, no correlation was found by VZV IFN-γ ELISPOT assay response among patients with STMc (Table 5).

**Table 3.** VZV-Specific Antibody Observed Response by gpELISA in Auto-HSCT Recipients and Patients With STMc, by HZ Confirmation (Per-Protocol Population)

gpELISA Units/mL^a^			Patients With Confirmed HZ				Patients Without Confirmed HZ			
			ZV_IN_		Placebo		ZV_IN_		Placebo	
			No.	Observed Response (90% CI)	No.	Observed Response (90% CI)	No.	Observed Response (90% CI)	No.	Observed Response (90% CI)
Auto-HSCT	Number of patients vaccinated: ZV_IN_, 554; placebo, 556									
	Baseline	GMT	28	81.3 (51.5–128.4)	75	138.3 (112.9–169.5)	346	109.2 (96.9–123.0)	309	108.3 (96.3–121.8)
	~28 days post–dose 4	GMT	4	152.2 (9.2–2521.6)	14	108.0 (64.1–181.7)	98	246.4 (189.6–320.2)	94	105.4 (82.3–135.1)
		GMFR^b^	4	1.62 (0.47–5.58)	14	0.71 (0.54–0.94)	97	2.15 (1.70–2.71)	92	1.02 (0.86–1.22)
STMc	Number of patients vaccinated: ZV_IN_, 1326; placebo, 1349									
	Baseline	GMT	21	177.1 (118.3–265.1)	57	144.1 (114.5–181.3)	1239	153.5 (146.1–161.4)	1230	151.2 (143.5–159.3)
	~28 days post–dose 4	GMT	14	307.9 (200.0–474.2)	40	141.0 (108.9–182.5)	943	293.8 (280.4–307.8)	971	158.4 (149.7–167.6)
		GMFR^b^	14	1.71 (1.34–2.18)	39	1.00 (0.93–1.08)	938	1.95 (1.87–2.02)	960	1.03 (1.00–1.06)

Abbreviations: auto-HSCT, autologous hematopoietic stem cell transplant; GMFR, geometric mean fold rise; gpELISA, glycoprotein enzyme-linked immunosorbent assay; HZ, herpes zoster; No., number of patients contributing to the immunogenicity analysis; STMc, solid tumor malignancies receiving chemotherapy; ZV_IN_, inactivated varicella zoster.

^a^Results for the gpELISA are reported as concentration of antibody in gpELISA units/mL.

^b^From day 1.

**Table 4.** VZV-Specific Cell-Mediated Observed Immune Response by IFN-γ ELISPOT Assay in Auto-HSCT Recipients, Patients With STMc, and Patients With HM, by HZ Confirmation (Per-Protocol Population)

IFN-γ ELISPOT Assay Count/10^6^ PBMCs^a^			Patients With Confirmed HZ				Patients Without Confirmed HZ			
			ZV_IN_		Placebo		ZV_IN_		Placebo	
			No.	Observed Response (90% CI)	No.	Observed Response (90% CI)	No.	Observed Response (90% CI)	No.	Observed Response (90% CI)
Auto-HSCT	Number of patients vaccinated: ZV_IN_, 186; placebo, 181									
	Baseline	GMC	9	33.2 (19.8–55.7)	20	54.5 (30.0–99.0)	119	30.7 (23.2–40.7)	109	30.8 (23.7–40.0)
	~28 days post– dose 4	GMC	11	30.2 (12.7–71.7)	19	5.9 (2.9–12.4)	91	68.7 (48.9–96.5)	97	11.7 (8.7–15.6)
		GMFR^b^	9	1.34 (0.63–2.87)	18	0.22 (0.12–0.39)	89	3.19 (2.23–4.57)	96	0.61 (0.44–0.85)
STMc	Number of patients vaccinated: ZV_IN_, 232; placebo, 246									
	Baseline	GMC	2	94.7 (NA)	7	55.5 (24.9–123.8)	175	64.3 (53.3–77.7)	194	68.9 (57.9–82.0)
	~28 days post– dose 4	GMC	3	301.3 (202.4–448.5)	4	56.5 (11.3–281.8)	165	161.6 (137.5–189.9)	167	79.8 (67.2–94.7)
		GMFR^b^	3	3.44 (1.69–6.98)	3	0.81 (0.40–1.65)	153	3.04 (2.49–3.72)	158	1.36 (1.16–1.61)
	Number of patients vaccinated: ZV_IN_, 231; placebo, 247									
HM	Baseline	GMC	5	16.3 (1.9–141.4)	2	121.1 (NA)	168	43.8 (34.5–55.7)	159	46.7 (36.6–59.7)
	~28 days post– dose 4	GMC	4	53.8 (5.2–557.8)	5	9.7 (0.9–105.6)	131	105.0 (83.4–132.3)	143	48.6 (38.0–62.2)
		GMFR^b^	4	1.38 (0.04–50.07)	5	0.75 (0.33–1.71)	124	2.20 (1.82–2.66)	132	1.03 (0.88–1.20)

Abbreviations: auto-HSCT, autologous hematopoietic stem cell transplant; GMC, geometric mean count; GMFR, geometric mean fold rise; HM, hematologic malignancies; IFN-γ ELISPOT, interferon-γ enzyme-linked immunospot; No., number of patients contributing to the immunogenicity analysis; NA, not available; PBMCs, peripheral blood mononuclear cells; STMc, solid tumor malignancies receiving chemotherapy; ZV_IN_, inactivated varicella zoster.

^a^Results from the IFN-γ ELISPOT assay are expressed as the frequency of spot-forming cells per million PBMCs.

^b^From day 1.

**Table 5.** Statistical Analysis of Immunogenic Response as a Correlate of Protection Against HZ, Using Cox Model in Auto-HSCT Recipients (VZV gpELISA Response), Patients With STMc (VZV gpELISA Response and VZV IFN-γ ELISPOT Assay Response), and Patients With HM (VZV IFN-γ ELISPOT Assay Response) in the Per-Protocol Population

gpELISA Units/mL^a^	Auto-HSCT^b^		STMc		HM	
	Point Estimate HR (95% CI)	*P* Value	Point Estimate HR (95% CI)	*P* Value	Point Estimate HR (95% CI)	*P* Value
Vaccine effect on HZ without adjustment for VZV gpELISA^c^	0.360 (0.25–0.51)	<.001	0.364 (0.22–0.59)	<.0001	—	—
Vaccine effect on HZ with adjustment for VZV gpELISA^d^	0.364 (0.24–0.56)	<.001	0.378 (0.23–0.63)	.0002	—	—
Effect of VZV gpELISA (log-scale) on the risk of HZ^d^	1.037 (0.90–1.20)	.613	0.957 (0.78–1.18)	.6796	—	—
IFN-γ ELISPOT Assay Count/10^6^ PBMCs^e^	Auto-HSCT		STMc		HM	
	Point Estimate HR (95% CI)	*P* Value	Point Estimate HR (95% CI)	*P* Value	Point Estimate HR (95% CI)	*P* Value
Vaccine effect on HZ without adjustment for VZV IFN-γ ELISPOT assay^f^	—	—	0.364 (0.22–0.59)	<.0001	0.833 (0.59–1.18)	.3035
Vaccine effect on HZ with adjustment for VZV IFN-γ ELISPOT assay^g^	—	—	0.376 (0.10–1.47)	.1603	1.337 (0.38–4.76)	.6536
Effect of VZV IFN-γ ELISPOT assay (log-scale) on the risk of HZ^g^	—	—	1.011 (0.63–1.61)	.9622	0.713 (0.54–0.94)	.0171

Abbreviations: auto-HSCT, autologous hematopoietic stem cell transplant; gpELISA, glycoprotein enzyme-linked immunosorbent assay; HM, hematologic malignancies; HZ, herpes zoster; IFN-γ ELISPOT, interferon-γ enzyme-linked immunospot; PBMCs, peripheral blood mononuclear cells; STMc, solid tumor malignancies receiving chemotherapy.

^a^Results for the gpELISA are reported as concentration of antibody in gpELISA units/mL.

^b^For vaccine effect on HZ incidence, the treatment-by-immunogenicity response interaction was statistically significant (*P = *.049); this *P* value for the interaction was calculated based on the likelihood ratio test.

^c^Computed based on a Cox regression model that included time to HZ onset as the response variable and treatment group, age stratum, and expected duration of antiviral prophylaxis (for auto-HSCT recipients) as explanatory variables.

^d^Computed based on a Cox regression model that included time to HZ onset as the response variable and treatment group, age stratum, expected duration of antiviral prophylaxis (for auto-HSCT recipients), and the natural log-transformed VZV gpELISA as time-varying explanatory variables.

^e^Results from the IFN-γ ELISPOT assay are expressed as the frequency of spot-forming cells per million PBMCs.

^f^Computed based on a Cox regression model that included time to HZ onset as the response variable and treatment group, age stratum, and HM immunocompromised stratum (for patients with HM) as explanatory variables.

^g^Computed based on a Cox regression model that included time to HZ onset as the response variable and treatment group, age stratum, and HM as explanatory variables.

## DISCUSSION

Defects in T-cell immunity increase the risk for HZ [1]. The gpELISA [21], which measures T-cell-dependent antibody responses, was shown in clinical studies of zoster vaccine to correlate with protection against HZ in healthy adults aged 50 years and older [24, 25]. At the time the phase 3 studies of ZV_IN_ were conducted, it was unknown if the same relationship between gpELISA and VE_HZ_ would be seen in immunocompromised patients. Therefore, gpELISA and VZV IFN-γ ELISPOT assay—a direct measure of T-cell immunity [22]—were incorporated into the phase 3 program. Two phase 3 studies were performed and demonstrated that ZV_IN_ was associated with a similar magnitude of efficacy among auto-HSCT recipients (estimated VE_HZ_ of 63.8%; 95% CI, 48.4–74.6) [7] and patients with STMc (estimated VE_HZ_ of 63.6%; 97.5% CI, 36.4–79.1) [8].

ZV_IN_ elicited higher VZV-specific responses vs placebo across different immunocompromised populations in the 2 phase 3 clinical efficacy studies described here. With regard to VZV-specific antibody responses measured by gpELISA, ZV_IN_ elicited a ~2-fold higher estimated GMFR ratio between ZV_IN_ and placebo at ~28 days post–dose 4 in auto-HSCT recipients and patients with STMc. Results of gpELISA from the present study in patients with STMc are similar to previous findings observed in 55 patients with STMc enrolled in V212-002 [17]. With respect to VZV-specific cell-mediated responses measured by the IFN-γ ELISPOT assay, ZV_IN_ elicited a ~2–5-fold higher estimated GMFR ratio between ZV_IN_ and placebo at ~28 days post–dose 4 across different immunocompromised populations. Among auto-HSCT recipients, the estimated GMFR ratio between treatment groups remained high at 1 and 2 years post–dose 4 (4.12 and 3.32, respectively). These results were similar to those observed in auto-HSCT recipients, patients with STMc (n = 56), and patients with HM (n = 60) enrolled in V212-002 [17].

The immunogenicity findings of ZV_IN_ described here are consistent with those observed with zoster vaccine live in a nonimmunocompromised patient population. In the zoster vaccine live shingles prevention study, conducted in 38_ _546 patients aged ≥60 years, cell-mediated immunity, assessed by IFN-γ ELISPOT assay, and humoral immunity, assessed by gpELISA, were significantly increased in patients receiving live attenuated VZV vaccine measured at 6 weeks after vaccination compared with those receiving placebo (IFN-γ ELISPOT: 70.1 vs 31.7 GMC; gpELISA: 471.3 vs 292.3 GMT), and the increase in cell-mediated immunity persisted for 3 years of follow-up [26]. Similarly, in the zoster vaccine live efficacy and safety trial, conducted in 22_ _439 patients 50–59 years of age, humoral immunity, assessed by gpELISA, was significantly increased, with a GMFR of 2.3 [27]. In both studies, a specific level for any immune response that was predictive of protection against HZ was not identified.

The efficacy results from trials V212-001 and V212-011 demonstrated ZV_IN_ vaccine efficacy in the prevention of HZ and HZ-related complications in auto-HSCT recipients and patients with STMc but not in patients with HM [7, 8]. The immunogenicity data presented here support an immune mechanism for ZV_IN_ vaccine efficacy in auto-HSCT recipients and patients with STMc, although a statistical correlation between immunogenicity and protection against HZ was not demonstrated. In fact, the IFN-γ ELISPOT assay GMCs observed ~28 days post–dose 4 in patients with STMc who received ZV_IN_ and subsequently developed HZ were higher than those observed in patients with STMc in the ZV_IN_ group who did not develop HZ. However, in this population that may have received chemotherapy after receiving 4 doses of ZV_IN_, measurement of immunity at ~28 days post–dose 4 may not accurately reflect immunity before the development of HZ. Waning of cell-mediated immunity due to chemotherapy could subsequently increase HZ risk, which is thought to depend on cell-mediated immunity at the time that latent VZV reactivates [1]. Interestingly, in patients with HM, positive VZV-specific cell-mediated responses measured by IFN-γ ELISPOT assay were observed in the ZV_IN_ group but did not translate into ZV_IN_ vaccine efficacy. These unexpected findings point to the importance of assessing vaccine clinical efficacy, as well as immunogenicity, to understand the protective potential of investigational HZ vaccines or established HZ vaccines administered to new patient populations. It is possible that immunogenicity measured by the assays reported here may not predict vaccine efficacy in certain immunocompromised patient populations or for all vaccines.

Long-term immunogenicity data assessed at 1 and 2 years post–dose 4 in auto-HSCT recipients revealed a decline in humoral immunity in the ZV_IN_ group, while cell-mediated immunity remained sustained. VZV-specific antibody responses by gpELISA in placebo-treated auto-HSCT recipients at 1 to 3 years post–dose 4 were lower than baseline values, a phenomenon previously seen in phase 1 testing [17], due to the highly immunosuppressive nature of the transplantation procedure performed after the baseline time point. As expected, VZV-specific cell-mediated immunity in the placebo group improved over the 2-year follow-up period of this study, as immunity has been shown to be restored following auto-HSCT procedures [28]. ZV_IN_ elicited higher cell-mediated responses compared with placebo up to the 2-year post–dose 4 time point.

A limitation of the V212-011 trial is that humoral immunity and cell-mediated immunity were not measured at 1 year post–dose 4; therefore, long-term immunogenicity data in patients with STMc and HM are not available. Although these are the first large vaccine trials conducted in these patient groups, the number of patients enrolled in both the V212-001 and V212-011 trials who were eligible to be included in the immunogenicity analysis was small. This precluded a robust analysis of the association between immune response and the risk of HZ. In trial V212-001, most exclusions from the immunogenicity analyses were due to use of prohibited concomitant medications, as expected in this patient population. Another limitation is that the Cox proportional hazards model used for the immunogenicity analyses did not include postrandomization use of immunosuppression. One key difference of the transplant population studied in the current trial vs the trials in immunocompetent patients is the relatively high proportion of patients with “nonvalid assays” in the ELISPOT assay (37 of 188 ZV_IN_ recipients [20%] and 39 of 182 placebo recipients [21%]) (Supplementary Figure 1). The observed effect may be due to the high level of immunosuppression, which is a well-known phenomenon among transplant recipients [29–31].

In summary, these 2 phase 3 trials demonstrated that ZV_IN_ elicits cellular immune responses when measured by IFN-γ ELISPOT assay in the 3 immunocompromised populations examined: auto-HSCT recipients, patients with STMc, and patients with HM. Although our immunogenicity data are consistent with the ZV_IN_ clinical efficacy in HZ prevention in auto-HSCT recipients and patients with STMc, demonstrated in the same studies, the immunogenicity data we report in patients with HM did not translate into clinical efficacy in HZ prevention.

## Supplementary Data

Supplementary materials are available at *Open Forum Infectious Diseases* online. Consisting of data provided by the authors to benefit the reader, the posted materials are not copyedited and are the sole responsibility of the authors, so questions or comments should be addressed to the corresponding author.

ofaa172_suppl_Supplementary_Figure_1

ofaa172_suppl_Supplementary_Figure_2

ofaa172_suppl_Supplementary_Material
