# Supplementary figures and images for: Immunogenicity of Inactivated Varicella Zoster Vaccine in Autologous Hematopoietic Stem Cell Transplant Recipients and Patients With Solid or Hematologic Cancer
Source: Open Forum Infect Dis. 2020 Jun 2;7(7):ofaa172. doi: 10.1093/ofid/ofaa172 (PMC7336559; doi:10.1093/ofid/ofaa172)

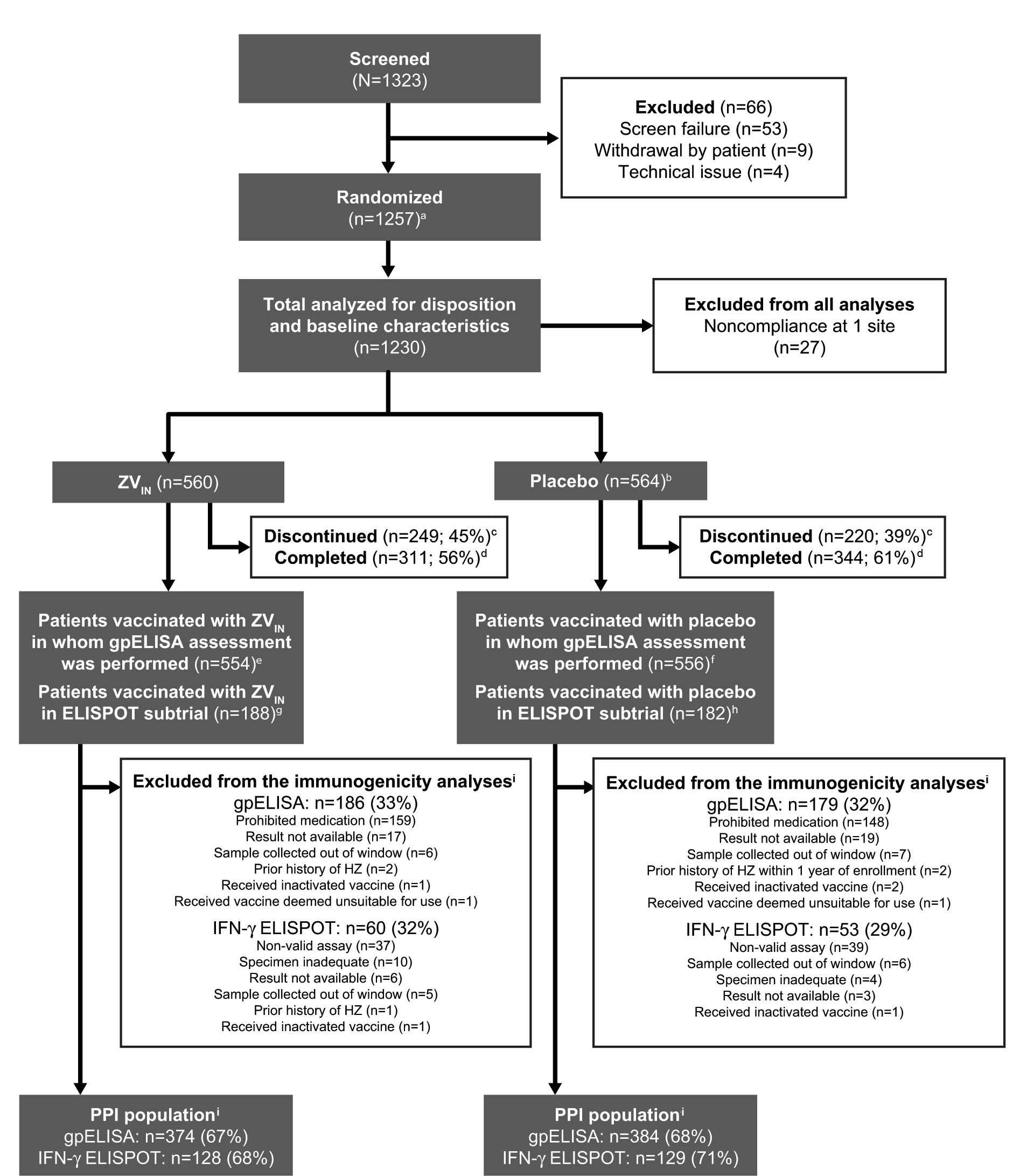

Supplement: ofaa172_suppl_Supplementary_Figure_1 [file ofaa172_suppl_supplementary_figure_1.png]

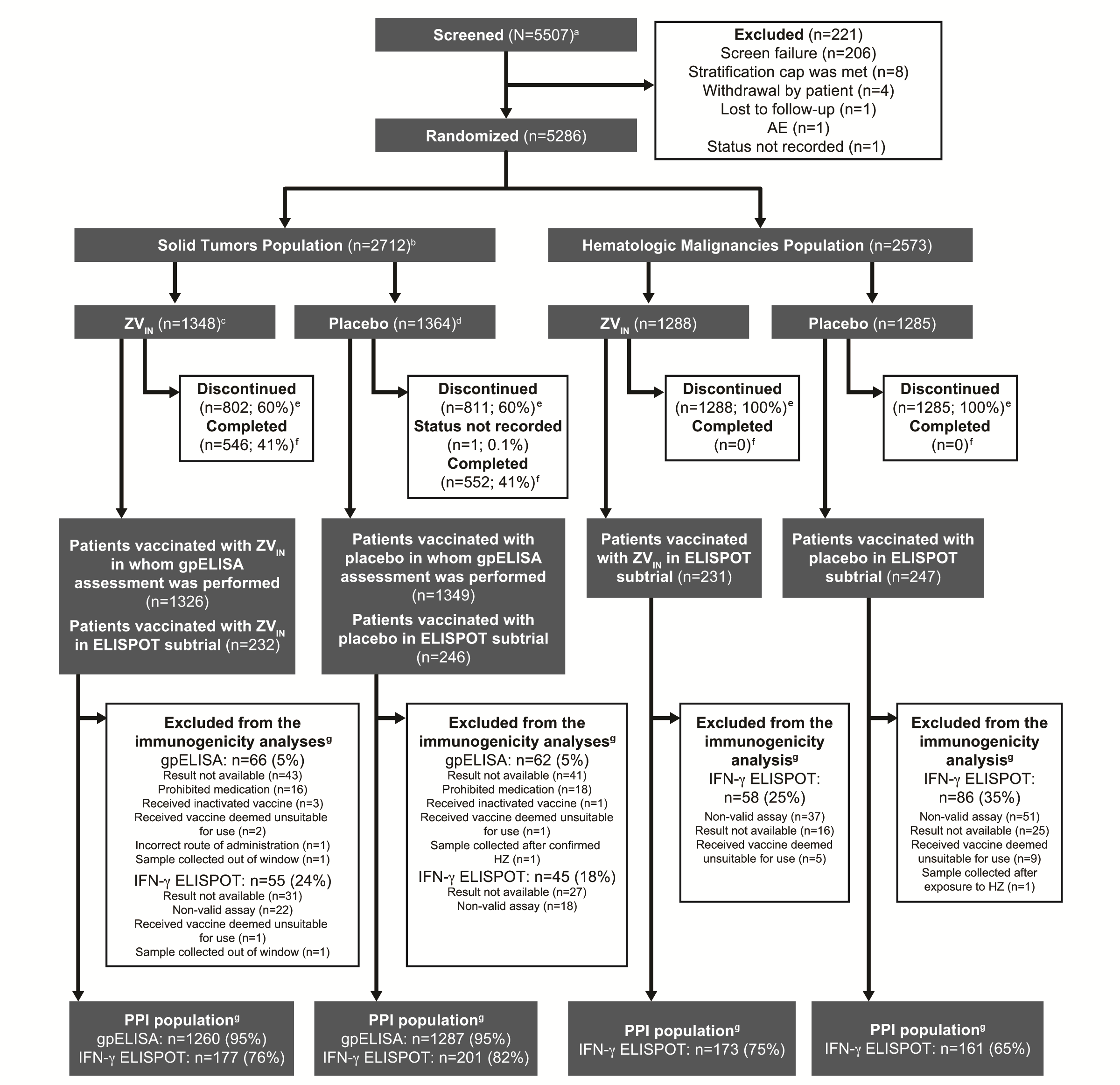

Supplement: ofaa172_suppl_Supplementary_Figure_2 [file ofaa172_suppl_supplementary_figure_2.png]
