# Supplementary material for: Immunogenicity of Inactivated Varicella Zoster Vaccine in Autologous Hematopoietic Stem Cell Transplant Recipients and Patients With Solid or Hematologic Cancer
Source: Open Forum Infect Dis. 2020 Jun 2;7(7):ofaa172. doi: 10.1093/ofid/ofaa172 (PMC7336559; doi:10.1093/ofid/ofaa172)
Supplement: ofaa172_suppl_Supplementary_Material [file ofaa172_suppl_supplementary_material.docx]

**Supplementary Material**

**V212 (ZV_IN_) Protocol 001 Trial Group**

Argentina: M.O. Dictar, P. Bonvehi, A.C. Basso, S. Cerana

Australia: J. Sasadeusz, P. Campbell, G. Playford

Belgium: J. Maertens, D. Selleslag, R. Schots, X. Poire, K. Theunissen, E. Willems

Brazil: J.F.C. Camargo, L. Maria Fogliatto, R.S. Alves, Rodrigo, N.S. Castro

Canada: F. Courture, A. McGeer, M. Miller

Colombia: J.F. Combariza, J.D. Velez, C.L. Sossa

Croatia: D. Nemet, S. Ostojic Kolonic

Czech Republic: L. Jebavy, J. Novak, D. Pohlreich, J. Mayer

Ecuador: B. Maldonado

France: L. Karlin, T. Gastinne, O. Launay

Germany: O.A. Cornely, D. Teschner, W. Heinz, M. Haenel, M. Kaufmann, M. Verbeek, G. Wulf, J. Panse, H.A. Duerk

Israel: A. Nagler, F. Aviv, M. Yeshurun, S. Grisariu

Italy: A. Velardi, F. Onida, A. Rambaldi, P. Corradini, A. Bosi, G. Martinelli

Korea: S.S. Yoon, J.W. Cheong, S.J. Kim, D.G. Lee

Lithuania: I. Trociukas

Mexico: A.D. Gomez

Netherlands: M.J. Wondergem, P.F. Ypma

Panama: E. Fanilla

Peru: M.D.C. Moreno Larrea

Portugal: C. Geraldes, R.B. Ferreira, M.M. Abecasis

Puerto Rico: J. Castro

Russian Federation: B.V. Afanasyev, A.Y. Zaritskiy, I.V. Kruchkova

Spain: I. Jarque Ramos, C. Solano Vercet, B. Aguado Bueno

Sweden: P. Ljungman, H. Cherif, K. Vaht

United Kingdom: E. Kanfer, A. Parker, D.W. Milligan, G. Cook

United States: F.R. Betts, J.M. Brown, L. Akard, S.A. Pergam, I. Braunschweig, M.P. Carroll, P.H. Chandrasekar, R. Collins, B. Cooper, M. Craig, M.J. Guarino, J.W. Hiemenz, L.M. Klein, O.I. Krijanovski, J.F. Leis, K.M. Mullane, G.A. Papanicolaou, V. Roy, G.B. Selby, J.M. Sloan, L.M. Strasfeld, M. Styler, E.A. Vance, P Flomenberg, S. Yanovich, S.J. Lawrence, W. Rybka, K. Sullivan, K.P. High, J. Mehta, E.D. Ball, C. Freytes, W. Tse, C. Bachier, H. Safah, N. D'Cunha, S. Shoham, S. Nathan, G.M. Segal, D. Winston, M.L. Donato, B.I. Mattar, J.P. Lynch, W.L. Longo, M.C. Hall, N. Janakiraman, L. Kaminer, M.W. Schuster, L.M. Isola, K. Komanduri, D. Salzman, K. Kane, A. Freifeld, J. Essell, M. Lill

**V212 (ZV_IN_) Protocol 011 Trial Group**

Argentina: S. Cerana, M.O. Dictar, P. Bonvehi, J.P. Tregnaghi, L. Fein

Australia: D. Ashley, M. Singh, T. Hayes, G. Playford, O. Morrissey

Austria: J. Thaler, T. Kuehr, R. Greil, M. Pecherstorfer

Belgium: L. Duck, K. Van Eygen, M. Aoun, B. De Prijck

Brazil: F.A. Franke, C.H.E. Barrios, A.V.A. Mendes, S.V. Serrano, R.F. Garcia, F. Moore, J.F.C. Camargo, L.A. Pires, R.S. Alves

Bulgaria: A. Radinov, K. Oreshkov, V. Minchev, A.I. Hubenova, T. Koynova, I. Ivanov, B. Rabotilova, V. Minchev, P.A. Petrov, P. Chilingirov, S. Karanikolov, J. Raynov

Canada: D. Grimard, S. McNeil, D. Kumar, L.M. Larratt, K. Weiss, R. Delage, F.J. Diaz-Mitoma, P.O. Cano, F. Couture

Chile: P. Carvajal, R. Torres Ulloa, P. Fardella, C. Caglevic, C. Rojas, E. Orellana, P Gonzalez, A. Acevedo

Colombia: A. Yepes, K.M. Galvez, M.E. Gonzalez, S. Franco, J.G. Restrepo, C.A. Rojas, C. Bonilla, L.E. Florez, A.V. Ospina, R. Manneh

Croatia: R. Zorica, D.V. Vrdoljak, M. Samarzija

Czech Republic: L. Petruzelka, J. Vydra, J. Mayer, D. Cibula, J. Prausova

Ecuador: G. Paulson, M. Ontaneda

Estonia: K. Palk, A. Vahlberg, R. Rooneem

France: F. Galtier, D. Postil, F. Lucht, F. Laine, O. Launay, H. Laurichesse, X. Duval

Germany: O.A. Cornely, B. Camerer, J. Panse, M. Zaiss, H.-G. Derigs, H. Menzel, M. Verbeek

Greece: V. Georgoulias, D. Mavroudis, A. Anagnostopoulos, E. Terpos

Honduras: D. Cortes, J. Umanzor, S. Bejarano, R.W. Galeano

Hong Kong: R.S.M. Wong, P. Hui

Italy: P. Pedrazzoli, L. Ruggeri, F. Aversa, A. Bosi, G. Gentile, A. Rambaldi, A. Contu, A. Pazzola

Jordan: L. Marei, A. Abbadi, W. Hayajneh

Lebanon: J. Kattan, F. Farhat, G. Chahine

Lithuania: J. Rutkauskiene

Mexico: L.J. Marfil Rivera, Y.A. Lopez Chuken, H. Franco Villarreal, J. Lopez Hernandez

New Zealand: H. Blacklock

Panama: R.I. Lopez

Peru: R. Alvarez, M. Gomez A, S. Quintana T, M.D.C. Moreno Larrea, J. Zorrilla S, E. Alarcon, C.A. Samanez F

Philippines: P.B. Caguioa, B.J. Tiangco

Puerto Rico: E.M. Mora, R.D. Betancourt-Garcia, D. Hallman-Navarro, L.J. Feliciano-Lopez, H.A. Velez-Cortes, F. Cabanillas

Romania: D.E. Ganea, T.E. Ciuleanu, D.G. Ghizdavescu, L. Miron, C.L. Cebotaru, C.I. Cainap, R. Anghel

Russian Federation: M.V. Dvorkin, O.A. Gladkov, N.V. Fadeeva, A.A. Kuzmin, O.N. Lipatov, I.I. Zbarskaya, F.S. Akhmetzyanov, I.V. Litvinov, B.V. Afanasyev, M. Cherenkova, D. Lioznov, I.A. Lisukov, Y.A. Smirnova, S. Kolomietz

Saudi Arabia: H. Halawani

Singapore: Y.T. Goh

Slovakia: L. Drgona, J. Chudej, M. Matejkova, M. Reckova

South Africa: B.L. Rapoport, W.M. Szpak, D.R. Malan, N. Jonas

South Korea: C.W. Jung, D.G. Lee, S.S. Yoon

Spain: J. Lopez Jimenez, I. Duran Martinez, J.F. Rodriguez Moreno, C. Solano Vercet, R. de la Camara, M. Batlle Massana

Taiwan: S.-P. Yeh, C.-Y. Chen, H.-H. Chou, C.-M. Tsai, C.-H. Chiu

Thailand: N. Siritanaratkul, L. Norasetthada, V. Sriuranpong, K. Seetalarom

Turkey: H. Akan, F. Dane, M.A. Ozcan, G.H. Ozsan, S.F. Kalayoglu Besisik, A. Cagatay, S. Yalcin

United Kingdom: A. Peniket

United States: K.M. Mullane, S.R. Dakhil, K. Sivarajan, J.J.-G. Suh, A. Sehgal, F. Marquez, E.G. Gomez, M.R. Mullane, W.L. Skinner, R.J. Behrens, D.R. Trevarthen, M.A. Mazurczak, E.A. Lambiase, C.A. Vidal, S.Y. Anac, G.A. Rodrigues, B. Baltz, R. Boccia, M.S. Wertheim, C.S. Holladay, D. Zenk, W. Fusselman, J.L. Wade III, A.J. Jaslowski, J. Keegan, M.O. Robinson, R.S. Go, J. Farnen, B. Amin, D. Jurgens, G.F. Risi Jr, P.G. Beatty, T. Naqvi, S. Parshad, V.L. Hansen, M. Ahmed, P.D. Steen, S. Badarinath, A. Dekker, M.A. Scouros, D.E. Young, W. Graydon Harker, S.D. Kendall, M.L. Citron, S. Chedid, J.G. Posada Jr, M.K. Gupta, S. Rafiyath, J. Buechler-Price, S. Sreenivasappa, C.H. Chay, J.M. Burke, S.E. Young, A. Mahmood, J.W. Kugler, G. Gerstner, J. Fuloria, N.D. Belman, R. Geller, J. Nieva, B.P. Whittenberger, B.M.Y. Wong, T.P. Cescon, G. Abesada-Terk Jr, M.J. Guarino, A. Zweibach, E.N. Ibrahim, G. Takahashi, M.A. Garrison, R.B. Mowat, B.S. Choi, I.A. Oliff, J. Singh, K.A. Guter, K. Ayrons, K.M. Rowland, S.J. Noga, S.B. Rao, A. Columbie, M.T. Nualart, G.R. Cecchi, L.T. Campos, M. Mohebtash, M.R. Flores, R. Rothstein-Rubin, B.M. O’Connor, G. Soori, M. Knapp, F.G. Miranda, B.W. Goodgame, M. Kassem, R. Belani, S. Sharma, T. Ortiz, H.L. Sonneborn, A.B. Markowitz, D. Wilbur, E. Meiri, V.S. Koo, H.S. Jhangiani, L. Wong, S. Sanani, S.J. Lawrence, C.M. Jones, C. Murray, C. Papageorgiou, J.S. Gurtler, J.L. Ascensao, K. Seetalarom, M.L. Venigalla, M. D’Andrea, C. De Las Casas, D.J. Haile, F.U. Qazi, J.L. Santander, M.R. Thomas, V.P. Rao, M. Craig, R.J. Garg, R. Robles, R.M. Lyons, R.K. Stegemoller, S. Goel, S. Garg, P. Lowry, C. Lynch, B. Lash, T. Repka, J. Baker, B.S. Goueli, T.C. Campbell, D.A. Van Echo, Y.J. Lee, E.A. Reyes, F.M. Senecal, G. Donnelly, P. Byeff, R. Weiss, T. Reid, E. Roeland, A. Goel, D.M. Prow, D.S. Brandt, H.G. Kaplan, J.E. Payne, M.J. Boeckh, P.J. Rosen, R.R. Mena, R. Khan, R.F. Betts, S.A. Sharp, V.A. Morrison, D. Fitz-Patrick, J. Congdon, N. Erickson, R. Abbasi, S. Henderson, A. Mehdi, E.J. Wos, E. Rehmus, L. Beltzer, R.A. Tamayo, T. Mahmood, A.C. Reboli, A. Moore, J.M. Brown, J. Cruz, D.P. Quick, J.L. Potz, K.W. Kotz, M. Hutchins, N.M. Chowhan, Y.D. Devabhaktuni, P. Braly, R.A. Berenguer, S.C. Shambaugh, T.J. O’Rourke, W.A. Conkright, C.F. Winkler, F.E.K. Addo, J.P. Duic, K.P. High, M.E. Kutner, R. Collins, D.R. Carrizosa, D.J. Perry, E. Kailath, N. Rosen, R. Sotolongo, S. Shoham, T. Chen

**Supplementary Methods**

**Vaccine Description**

The inactivated VZV vaccine and placebo used in this trial were manufactured by Merck & Co. Inc. (West Point, PA, USA). The vaccine and placebo were sterile and lyophilized. Placebo was the inactivated VZV vaccine stabilizer with no virus antigen. The individual inactivated VZV vaccine (an inactivated version of the zoster vaccine live vaccine) and placebo doses were packaged in single-dose, 3-mL glass vials and shipped to the trial sites at room temperature (defined as 20°C to 25°C [68°F to 77°F]) or refrigerated with a contained temperature- monitoring device. Upon receipt, the vaccine and placebo were refrigerated and stored at 2°C to 8°C (35°F to 46°F) or colder until reconstitution. The diluent was 0.7 mL of sterile water without preservatives or other substances that might inactivate the vaccine, and was shipped and stored at room temperature or at 2°C to 8°C (35°F to 46°F). The vaccine/placebo was reconstituted immediately before administration, using only the diluent that was provided by the Sponsor, by withdrawing the entire contents of the diluent vial into a syringe, injecting the entire contents of the syringe into the vial lyophilized vaccine (~0.7 mL), and gently agitating the mixture thoroughly. A total of 0.5 mL of reconstituted vaccine/placebo (visually indistinguishable from each other) was withdrawn and administered to each patient enrolled in the trial via subcutaneous injection.

**Supplementary Figure 1.** Disposition of autologous HSC transplant recipients.

gpELISA, glycoprotein ELISA; HZ, herpes zoster; IFN-γ ELISPOT, IFN-gamma enzyme-linked immunospot assay; PPI, per-protocol immunogenicity; ZV_IN_, inactivated varicella zoster.

^a^Twenty-seven participants enrolled at a single trial site were excluded due to Good Clinical Practice compliance issues.

^b^One patient was randomized to receive placebo but received 1 dose of ZV_IN_ and was therefore excluded from the immunogenicity analysis.

^c^Patients who discontinued the study were those who died or those who withdrew from the study prior to study completion due to adverse events, disease progression, patient’s decision, physician’s decision, Sponsor’s decision, missed follow-up, and protocol violation. These patients contributed to the immunogenicity analyses only during the time they were on the study.

^d^Patients who completed the study had to have completed the study close-out questionnaire at the end of the safety follow-up period, which was administered over the phone.

^e^Six patients did not receive ZV_IN_ and hence did not contribute to the gpELISA analysis.

^f^Eight patients did not receive placebo and hence did not contribute to the gpELISA analysis.

^g^Two patients did not receive ZV_IN_ and hence did not contribute to the IFN-γ ELISPOT analysis.

^h^One patient did not receive placebo and hence did not contribute to the IFN-γ ELISPOT analysis.

^i^At baseline.

**Supplementary Figure 2.** Disposition of patients with solid tumors receiving chemotherapy and patients with hematologic malignancies.

AE, adverse event; gpELISA, glycoprotein ELISA; HZ, herpes zoster; IFN-γ ELISPOT, IFN-gamma enzyme-linked immunospot assay; PPI, per-protocol immunogenicity; ZV_IN_, inactivated varicella zoster.

^a^Nineteen patients from a single trial site were excluded due to Good Clinical Practice compliance issues.

^b^One patient was randomized to ZV_IN_ but not vaccinated; the patient’s population was not recorded, and the patient was not included in any of the analyses.

^c^Twenty patients did not receive ZV_IN_, and 2 patients received a dose of placebo instead of ZV_IN_. These patients did not contribute to the gpELISA analysis.

^d^Fourteen patients did not receive placebo, and 1 patient received a dose of ZV_IN_ instead of placebo. These patients did not contribute to the gpELISA analysis.

^e^Patients who discontinued the study were those who died or those who withdrew from the study prior to study completion due to adverse events, disease progression, patient’s decision, physician’s decision, Sponsor’s decision, missed follow-up, and protocol violation. These patients contributed to the immunogenicity analyses only during the time they were on the study.

^f^Patients who completed the study had to have completed the study close-out questionnaire at the end of the safety follow-up period, which was administered over the phone.

^g^At baseline.

**Supplementary Table 1. Baseline Demographics of Auto-HSCT Recipients, Patients With STMc, and Patients With HM**

| Characteristics | Auto-HSCT Recipients | |
| --- | --- | --- |
|  | ZV_IN_  (n = 560) | Placebo  (n = 564) |
| **Mean age, years (SD)**  <50 years, n (%)  ≥50 years, n (%) | 54.1 (12.6)  158 (28.2)  402 (71.8) | 54.1 (12.2)  159 (28.2)  405 (71.8) |
| **Sex, n (%)**  Male  Female | 357 (63.8)  203 (36.3) | 360 (63.8)  204 (36.2) |
| **Underlying disease, n (%)**  Myeloma  Diffuse large B-cell lymphoma  Hodgkin lymphoma  Mantle cell lymphoma  Other non-Hodgkin lymphoma  Other | 238 (42.5)  57 (10.2)  45 (8.0)  43 (7.7)  33 (5.9)  144 (25.7) | 223 (39.5)  56 (9.9)  42 (7.4)  57 (10.1)  45 (8.0)  141 (25.0) |
| **Most frequent (in ≥50% of the patients) concomitant medications,^a^ n (%)** | n^b^ = 554 | n^b^ = 556 |
| Systemic antibacterials | 539 (97.3) | 543 (97.7) |
| Analgesics | 508 (91.7) | 511 (91.9) |
| Antiemetics and antinauseants | 506 (91.3) | 514 (92.4) |
| Antineoplastic agents | 503 (90.8) | 509 (91.5) |
| Drugs for acid-related disorders | 497 (89.7) | 510 (91.7) |
| Antivirals for systemic use | 494 (89.2) | 502 (90.3) |
| Corticosteroids for systemic use | 469 (84.7) | 475 (85.4) |
| Immunostimulants | 465 (83.9) | 471 (84.7) |
| Blood substitutes and perfusion solutions | 456 (82.3) | 467 (84.0) |
| Systemic antimycotics | 454 (81.9) | 466 (83.8) |
| Systemic antihistamines | 453 (81.8) | 448 (80.6) |
| Psycholeptics | 440 (79.4) | 463 (83.3) |
| Diuretics | 352 (63.5) | 345 (62.1) |
| GI disorder agents | 314 (56.7) | 313 (56.3) |
| Antithrombotic agents | 276 (49.8) | 287 (51.6) |
| Characteristics | Patients With STMc | |
|  | ZV_IN_  (n = 1348) | Placebo  (n = 1364) |
| **Mean age, years (SD)**  <50 years, n (%)  ≥50 years, n (%) | 57.6 (11.5)  299 (22.2)  1049 (77.8) | 57.7 (11.5)  320 (23.5)  1044 (76.5) |
| **Sex, n (%)**  Male  Female | 481 (35.7)  867 (64.3) | 472 (34.6)  892 (65.4) |
| **Underlying disease, n (%)**  Breast cancer  Colon, colorectal, and rectal cancer  Lung cancer  Ovarian cancer  Other | n^b^ = 1328  499 (37.6)  289 (21.8)  152 (11.4)  64 (4.8)  324 (24.4) | n^b^ = 1350  496 (36.7)  279 (20.7)  146 (10.8)  69 (5.1)  360 (26.7) |
| **Most frequent (in ≥50% of the patients) concomitant medications,^a^ n (%)**  Antineoplastic agents  Antiemetics and antinauseants  Systemic corticosteroids  Drugs for acid-related disorders  Analgesics | n^b^ = 1328  1292 (97.3)  996 (75.0)  972 (73.2)  803 (60.5)  737 (55.5) | n^b^ = 1350  1315 (97.4)  1021 (75.6)  981 (72.7)  815 (60.4)  754 (55.9) |
| Characteristics | Patients With HM | |
|  | ZV_IN_  (n = 1288) | Placebo  (n = 1285) |
| **Mean age, years (SD)**  <50 years, n (%)  ≥50 years, n (%) | 61.0 (14.9)  235 (18.2)  1053 (81.8) | 61.4 (14.5)  223 (17.4)  1062 (82.6) |
| **Sex, n (%)**  Male  Female | 760 (59.0)  528 (41.0) | 762 (59.3)  523 (40.7) |
| **Underlying disease, n (%)**  Chronic lymphocytic leukemia  Myeloma  Chronic myeloid leukemia  Non-Hodgkin lymphoma  Other | n^b^ = 1277  324 (25.4)  199 (15.6)  154 (12.1)  116 (9.1)  484 (37.9) | n^b^ = 1275  343 (26.9)  197 (15.5)  152 (11.9)  93 (7.3)  490 (38.4) |
| **Most frequent (in ≥50% of the patients) concomitant medications,^a^ n (%)**  Antineoplastic agents  Analgesics | n^b^ = 1277  702 (55.0)  785 (61.5) | n^b^ = 1275  682 (53.5)  755 (59.2) |
| Abbreviations: auto-HSCT, autologous HSC transplant; HM, hematologic malignancies; STMc, solid tumor malignancies receiving chemotherapy; ZV_IN_, inactivated varicella zoster.  ^a^After any vaccination through 28 days following the fourth vaccination dose.  ^b^Includes all patients randomized in the trial who received at least one dose of the vaccination. | | |
